# Hospital Sunday, 1919

**Published:** 1919-06-21

**Authors:** 


### The Hospital

The Workers' Newspaper of Administrative Medicine and Institutional Life, Administration, National Insurance and Health.

No. 1724, Vol. LXVI. SATURDAY, JUNE 21, 1919. PRICE TWOPENCE

### HOSPITAL SUNDAY, 1919.

The inhabitants of Greater London should individually and collectively put their hearts into the effort to raise at least £100,000 on Hospital Sunday, the 22nd instant. The active and devoted leadership of the Lord Mayor, the Bishop of London, the Archdeacon of London (Canon Holmes), and Mr. Robert Holland-Martin, Chairman of the Finance Committee of the Hospital Sunday Fund, with the co-operation of all the churches, should make this result a certainty. The leaders show that recent efforts have resulted in increasing individual church collections from £64 to £112, from £35 to £100, from £6 to £122, and from 17s. 6d. to £53. There are other examples which show an average increase of over 300 per cent. The Hospital Sunday Fund was never more ably administered, or more earnestly and unitedly worked for than it is to-day. The advertisements this year in the London newspapers are typical and convincing of the great energy and wonderful ability brought to bear on behalf of the Fund. Every resident within Greater London has a call, therefore, to give a thank-offering for the war work of the hospitals. The Metropolis of the Empire, through its hospitals, has nobly taken its part in providing for the wounded from the war. We hope that, following Birmingham's example, the places of worship on Sunday next will be filled with family groups, and that they may be seen marching to the churches, with grateful hearts, each bearing a gift to the Hospital Sunday Fund. Who amongst all us Londoners can there be, who will be able to share in the Peace rejoicings, will forget to give to the sick on Hospital Sunday, 1919?

